# Maternal Investment Is Positively Associated With the Presence of Extra‐Pair Offspring in a Socially Monogamous Songbird

**DOI:** 10.1002/ece3.71169

**Published:** 2025-03-20

**Authors:** Valerie N. Brewer, Samuel J. Lane, Isaac J. VanDiest, Karen E. Mabry, Kendra B. Sewall

**Affiliations:** ^1^ Department of Biological Sciences Virginia Tech Blacksburg Virginia USA; ^2^ Department of Biology New Mexico State University Las Cruces New Mexico USA; ^3^ Department of Biological Sciences and School of Neuroscience Virginia Tech Blacksburg Virginia USA

**Keywords:** extra‐pair paternity, maternal investment, parental care

## Abstract

Biparental care is common in socially monogamous avian species, but both partners may seek extra‐pair copulations (EPCs). The relative costs and benefits of EPCs between the sexes are likely complex, yet the implications of EPCs for parental care behavior have been examined predominantly in males. Not only could females benefit from EPCs, but females would have additional information about the likelihood of extra‐pair young (EPY) in their nest not available to their partners, which likely influences female behavior. We examined how the presence and abundance of EPY in a nest affect parental behavior in a socially monogamous songbird, song sparrows ( *Melospiza melodia* ). We predicted that females who mated outside the social pair would invest more in a clutch with a higher probability of EPY. We monitored nest visitation rates by male and female social partners as a proxy for parental investment and quantified extra‐pair paternity in 45 nests. Maternal visitation rates were higher in nests with EPY compared to nests without, while males did not adjust their investment in relation to the presence of EPY. These findings support our prediction that females who participated in EPC would invest more in the resulting offspring.

## Graphical abstract

We explored the relationship between maternal and paternal investment (nest visitation rates) and the paternity of nestlings in a wild, socially monogamous songbird. We found that maternal investment was positively associated with the presence, number, and proportion of extra‐pair nestlings, but there was no relationship between paternal investment and extra‐pair young. These findings support our prediction that females who participated in extra‐pair copulations would invest more in the resulting offspring.

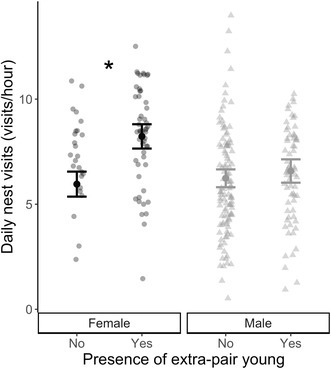

## 1 Introduction

Parental investment, including direct parental care, can have a strong influence on the survival and reproductive success of offspring (Møller and Thornhill 1998; Parish and Coulson 1998; Wendeln and Becker 1999). In many species of songbirds, both male and female parents provide care for offspring (Cockburn 2006). Most songbird species are socially monogamous as they form mate pairs during the breeding season; however, they are not genetically monogamous, as in these systems, both males and females may produce extra pair (EP) offspring (Griffith et al. 2002). Potential benefits of EP mating for males include the production of a larger number of offspring in exchange for relatively low effort and the distribution of offspring across more nests, mitigating the risk of clutch failure. However, the benefits for females may be more complex to discern. For females, EP copulations could provide direct benefits such as access to resources controlled by the EP male; for example, EP red‐winged blackbird ( *Agelaius phoeniceus* ) males have been observed to allow females that copulated with them to forage on their territory (Gray 1997). These EP copulations could also mitigate the risk of mating with an infertile partner (Sheldon 1994; Santema et al. 2020). Females may also directly benefit from an increase in cooperative behaviors from EP males towards the pair caring for his young (Eliassen and Jørgensen 2014), potentially contributing to the reduced brood failure observed in EP nests (Mennerat et al. 2018). For example, an EP male may provide defense against predators at nests where he has sired young (Gray 1997; Krams et al. 2022). Indirect benefits of EP copulations to the female in the form of genetic benefits to her offspring have also been hypothesized, such as increased genetic diversity or “better quality” genes in her offspring. These genetic benefits could increase the likelihood that her genes will be passed on if they improve the survival or reproductive success of the offspring; yet, evidence for these genetic benefits has been found to be weak at best (Akçay and Roughgarden 2007). If females benefit from EP copulations, they are expected to invest more in nests that have EP young (Burley 1988; Bluhm and Gowaty 2004; Loyau et al. 2007; Gowaty 2012).

Male parental care has been extensively examined in the context of EP paternity because the potential fitness cost of losing paternity in a male's own nest is intuitive: siring fewer young in the nest may reduce reproductive success for that breeding attempt if he does not gain paternity elsewhere. Across bird species, paternal investment has been negatively associated with the frequency of EP paternity (Moller and Birkhead 1993; Møller 2000; Arnold and Owens 2002; Ball et al. 2017; Søraker et al. 2023). Males tend to provide more paternal care when levels of EP paternity are lower and when biparental care is needed for successful reproduction. However, within species, the relationship between EP paternity and paternal care is less clear. Some studies find a reduction in male care with the presence or proportion of EPY (Morton et al. 1990; Dixon et al. 1994; Chuang‐Dobbs et al. 2001; Suter et al. 2009; Gow et al. 2019), some unexpectedly find increased male care with the presence of EPY (García‐Vigón et al. 2009; Du et al. 2015), and others find no relationship between these variables (Whittingham and Lifjeld 1995; Yezerinac et al. 1996; MacDougall‐Shackleton and Robertson 1998; Peterson et al. 2001; Li and Brown 2002; Dickinson 2003; Maguire and Safran 2010; Villavicencio et al. 2014; Barati et al. 2018; Cousseau et al. 2020; Gao et al. 2020; Li et al. 2021; Poblete et al. 2021). This range of observations suggests that some males are unable to discern offspring paternity (Lattore et al. 2019), that patterns of paternity do not vary predictably between breeding attempts, or that the risks of reducing care to the nest outweigh the costs of feeding unrelated young (Whittingham et al. 1992; Westneat and Sherman 1993; Whittingham and Dunn 2001).

In contrast to paternal care, maternal care in relation to EP paternity has received significantly less attention. While the response of males to EPY may depend in part on their ability to accurately assess paternity (Westneat and Sherman 1993; Whittingham and Dunn 2001), females should have more complete information about the paternity of their offspring than male social partners because females should know whether they participated in EPCs. This information disparity between social partners implies that females should have a higher capacity than males for adjusting their parental investment in response to EPY if EPCs are beneficial for the female. However, studies examining female modulation of care as a function of EPY in passerines also have mixed results, suggesting that this modulation of care may be context‐and species‐specific. Some have found that maternal investment was positively associated with the presence or proportion of EPY (Chuang‐Dobbs et al. 2001; Suter et al. 2009; McFarlane et al. 2010; Sakamoto et al. 2023) others found no relationship (Dixon et al. 1994; García‐Vigón et al. 2009; Maguire and Safran 2010; Gow et al. 2019; Li et al. 2021), and some even detected a negative relationship (Li and Brown 2002; Du et al. 2015). Thus, it remains unclear how widespread female modulation of care is across species.

It is often assumed that females' responses to EPY are related to compensation for behaviors of helpers, whether those are male social partners or helpers at the nest (e.g., EP mates, previous year offspring). The possibility that females modulate care based on the benefits they reap from EPY, independent of social partner or helper behavior, has received less consideration. For example, changes in female investment in response to EPY have been interpreted as compensation for altered provisioning behavior by the social male (Chuang‐Dobbs et al. 2001; Suter et al. 2009; Du et al. 2015) or for contributions from the EP male (Li and Brown 2002). In contrast, four studies found no modulation of female investment when EPY were present (Dixon et al. 1994; García‐Vigón et al. 2009; Maguire and Safran 2010; Gow et al. 2019; Li et al. 2021), and single study found that females increased their provisioning behavior when there were EPY in the nest, and males compensated for this by decreasing their provisioning rate (Sakamoto et al. 2023). Another study found that female investment in the form of egg volume increased with the number of EPY (McFarlane et al. 2010). Thus, there is evidence that females may modulate their investment in EPY, and that male investment may or may not vary with changes in female effort, across studies of diverse species and mating systems. Though compensation has been inferred in many studies, distinguishing between compensation and independent modulation of parental investment by females requires measuring male and female parental effort in a system in which the female's behavior can be clearly separated from the behaviors of other individuals. Such studies of female responses to EPY will provide insight into the costs and benefits of EP behavior for females.

Here we examined how the EP paternity status of a nest influenced parental investment by both male and female song sparrows ( *Melospiza melodia* ). Song sparrows are territorial, socially monogamous passerines with moderate levels of EP paternity (Hill et al. 2011; Sardell et al. 2011; Brouwer and Griffith 2019). Further, female provisioning behavior in our population during the time of this study is positively correlated with male provisioning (Lane et al. 2023), suggesting that females are not compensating for changes in male provisioning. Thus, we can separate the female response from compensation in this study, making the song sparrow a straightforward study species for examining parental behaviors. Female song sparrows have an informational advantage over their social males regarding their EP partners during a breeding attempt. Moreover, EPCs may benefit females indirectly by improving the fitness of their offspring (possibly through additive genetic variation (Sardell, Arcese and Keller, et al. 2012; Sardell, Arcese and Reid, et al. 2012; Reid and Wolak 2018)), so we predicted that females would invest more in a clutch that had a higher probability of extra‐pair young (EPY). Thus, we expected that maternal investment, measured as nest visitation rates using radio frequency identification (RFID), would be higher in nests with the presence of EPY. We note here and throughout that nest visitation, as a proxy for parental investment, may not reflect feeding frequency, but does reflect effort and thus parental investment in a brood. In contrast, we predicted that males, who may not have information about the potential occurrence of EPP, either would not modulate parental investment in relation to EPY or, if they can detect EPCs by females or EPY in the nest, would decrease visitation rates with decreasing paternity in the nest.

## 2 Methods

### 2.1 Study Sites and Subjects

To examine the effects of extra‐pair young on parental care, we studied wild song sparrows at 4 established field sites in Montgomery County, Virginia, USA; for detailed maps, photos, and descriptions, please see (Lane et al. 2024). All sites were within 13.5 km of each other. Song sparrows in our population typically have two breeding attempts during the breeding season, with four chicks per brood. Their nestling period lasts approximately 10 days (Friedmann 1943). All research was conducted under permits pre‐approved by Virginia Tech's Institutional Animal Care and Use Committee and was conducted under current state and federal scientific collecting permits.

From March to July 2018 and 2019, we searched for and monitored song sparrow nests until either the nestlings fledged or the nest failed (Martin and Geupel 1993; Lane et al. 2023). When nests were located, we identified the stage. If the nests were in build or lay, we checked them every 2 days until clutch confirmation (i.e., the same clutch size for two checks). If they were located after incubation, two eggs were candled from the clutch, and the incubation age was estimated using information from (Lokemoen and Koford 1996). Nests were opportunistically found throughout the season, so we were unable to control for brood number, but only one brood per individual was included.

Once we were able to estimate the incubation age, we returned to the nest on days 10–12 of incubation to install the RFID system at the nest and catch the parents. We developed the RFID/PIT tag monitoring systems following the methods established by Bridge and Bonter to monitor parental visitation (Bonter and Bridge 2011; Bridge et al. 2019; Farr et al. 2021). We then modified and installed the RFID system, consisting of an RFID reader and battery stored in a weatherproof container and an antenna made from spooled magnetic wire at each nest, following the methods in Lane et al. (2023). We captured parents to obtain blood samples for genetic analysis and to attach passive integrated transponder (PIT) tags for use in the identification and monitoring of nest attendance. To do this, we captured adult male and female song sparrows with mist nets from 05:00 to 11:15 h. We took a small blood sample via brachial venipuncture and banded adults with a unique color band and PIT tag (2.12 × 0 mm; CYNTAG Inc.; Item#: 601205–2248; all tags were significantly < 4% of the body mass of adult song sparrows). Briefly, we calculated the daily parental visitation rate (visits/h) as the number of visits by an individual divided by the number of hours that individual was active at the nest that day (time of last visit—time of first visit). Though visitation does not directly reflect the nature of parental care at the nest, adults had to be within *~*3 cm of the nest to trigger a visitation event, and such nest attendance does require parental time and energy. Additionally, previous studies have shown that nest visitation and feeding rates are highly correlated (McCarty 2002). After the RFID systems were installed, we continuously monitored the parental visitation rate 24 h a day from hatch until fledge, or until the nest failed (on average, 7 days of parental care). In total, we monitored maternal care across the nestling period at 12 nests, 5 without EPY and 7 with EPY, for a total of 25 (no EPY) and 51 days (EPY) of maternal care data across all nests. For paternal care, we monitored 25 nests, 16 without EPY, and 9 nests with EPY for a total of 111 and 64 days of paternal care, respectively.

When nestlings were near fledging (Days 8–10 of the nestling period), we took a small blood sample from all nestlings via brachial venipuncture. We did not sample nestlings younger than 8 days of age; as we note in the results, this could influence calculations of the proportion of EPY in a nest due to nestlings that did not survive to Day 8. Whole blood from nestlings and adults was blotted onto filter paper in the field and then stored in the lab in an airtight container with Drierite (Trudeau et al. 2007).

### 2.2 Paternity Assignment

We extracted genomic DNA from blood blots and genotyped individuals at 12 microsatellite loci developed for use with song sparrows (Sardell et al. 2010; Nietlisbach et al. 2015) following methods described by Brewer et al. (2020). Alleles were reviewed manually and were scored and binned using geneious prime 2019.0.4 (https://www.geneious.com). We used genepop 4.2 (Raymond and Rousset 1995; Rousset 2008) to test each locus for conformance to Hardy–Weinberg equilibrium and to determine if linkage disequilibrium (LD) existed between any pairs of loci. We used paternity exclusion by direct comparison of the genotypes of offspring and social parents within a nest to determine whether offspring were sired outside the social pair (Jones et al. 2010). We conducted paternity exclusion analysis for all nests for which we obtained a genotype from the social father and at least 1 nestling, a total of 45 nests. The number of genotyped offspring in a nest ranged from 1 to 4 (2.70 ± 0.21; mean ± 1 SE) primarily due to variation in brood size and/or brood reduction before sampling. An offspring was considered within‐pair if it had 0–1 mismatches with the social father's genotype and extra‐pair if there were > 1 mismatches with the social father, a standard practice in paternity exclusion (Jones et al. 2010). This estimator of the occurrence of extra‐pair paternity allows for the possibility of inconsistencies between parent and offspring due to genotyping error or mutation.

We genotyped 548 song sparrows: 379 adults and 169 offspring and performed paternity exclusion analysis only for the 117 offspring from 45 nests where the social male was genotyped. Sixteen of the 45 nests were found to have at least one extra‐pair offspring (35.6%) and 32 of 117 nestlings were extra‐pair (27.4%). Rates of EPP ranged from 0% (no identifiable EPY) to 100% (all EPY) within a nest. In four of the seven cases in which 100% of young were EPY, all nestlings had blood samples taken (broods ranging from 2 to 4 nestlings). The remaining three cases involved singleton nestlings, which potentially inflate the proportion of EPY in that nest, which is why we looked at both the number and proportion of nestlings.

### 2.3 Statistical Analysis

**FIGURE 1:**
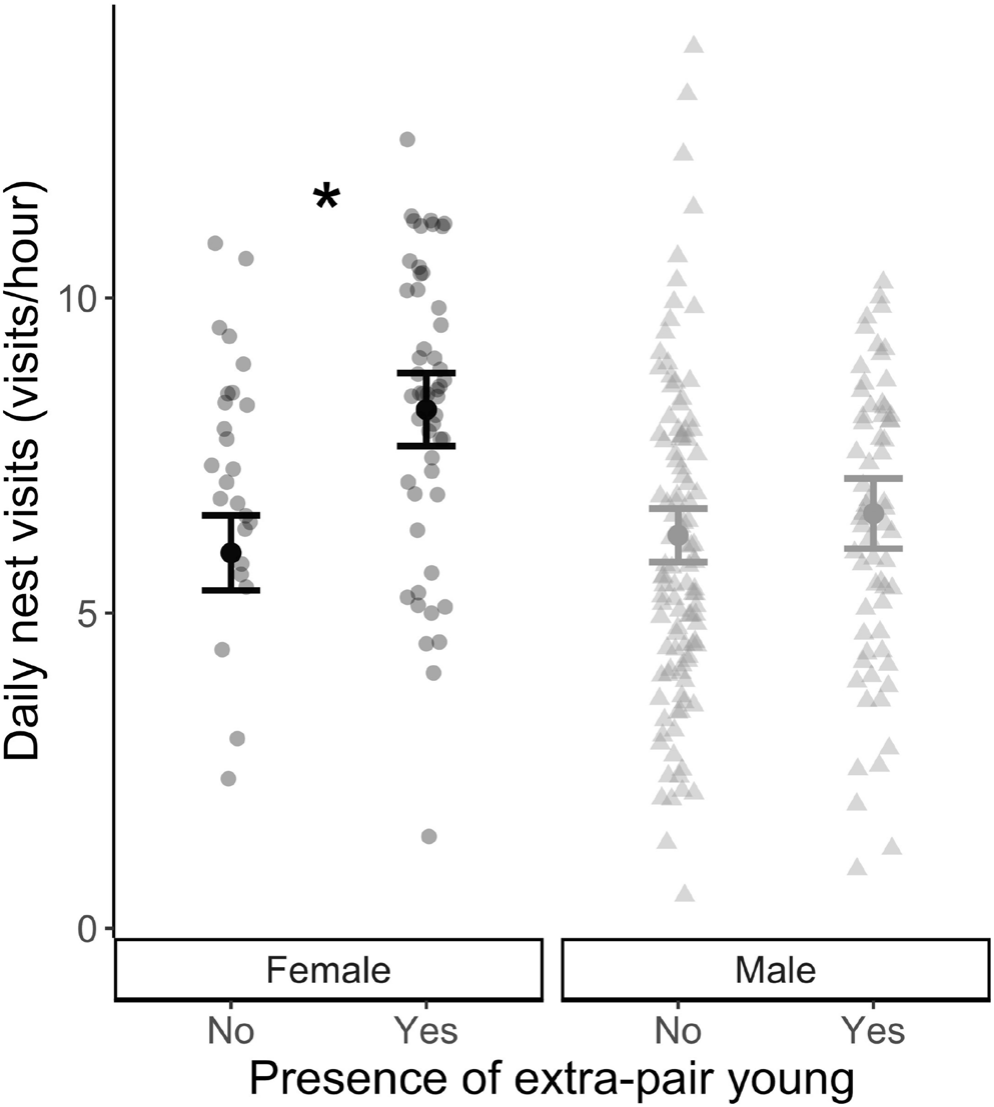
Parental visitation between nests with (black) and without (gray) EPY during the nestling period. The figure displays the model‐predicted mean ± SE over raw data points, each represents daily nest visitation rates for 12 females and 25 males. Significance is indicated by asterisk.

**FIGURE 2:**
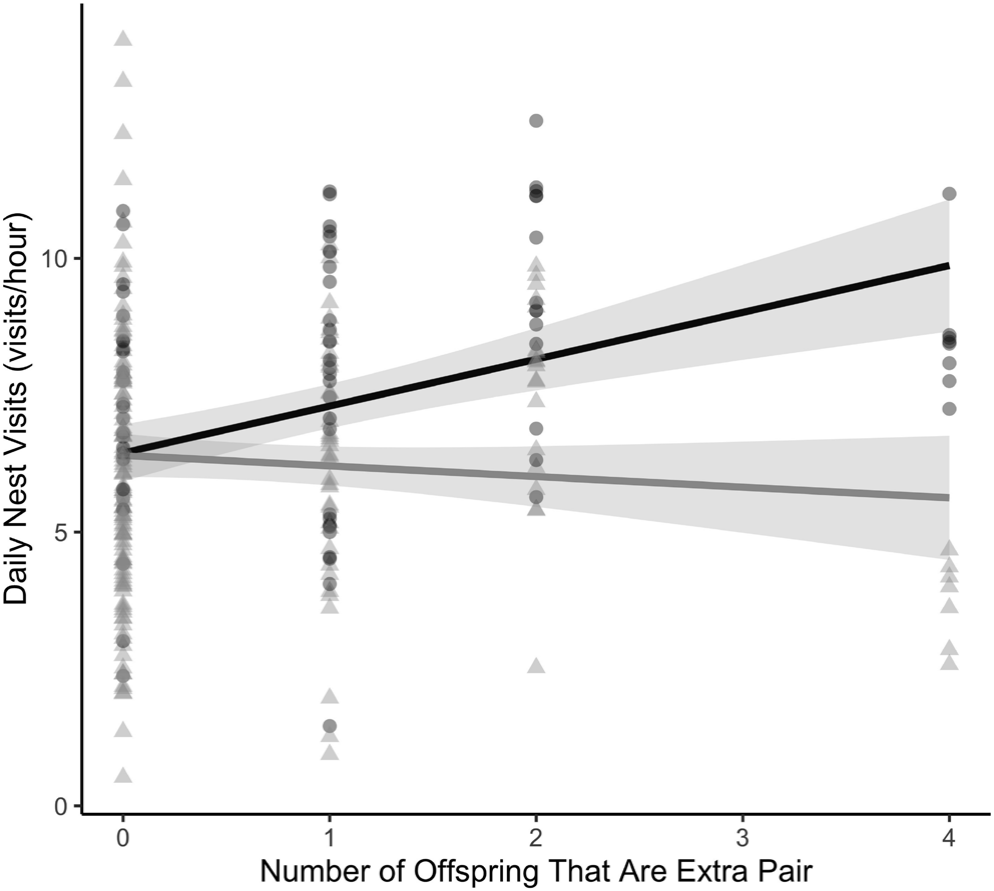
The relationship between maternal (black, circles) and paternal (gray, triangles) visitation rates and the number of extra‐pair young in the nest. Model predicted mean ± SE over raw data points, which each represents daily nest visitation rates for 12 females and 25 males.

**FIGURE 3:**
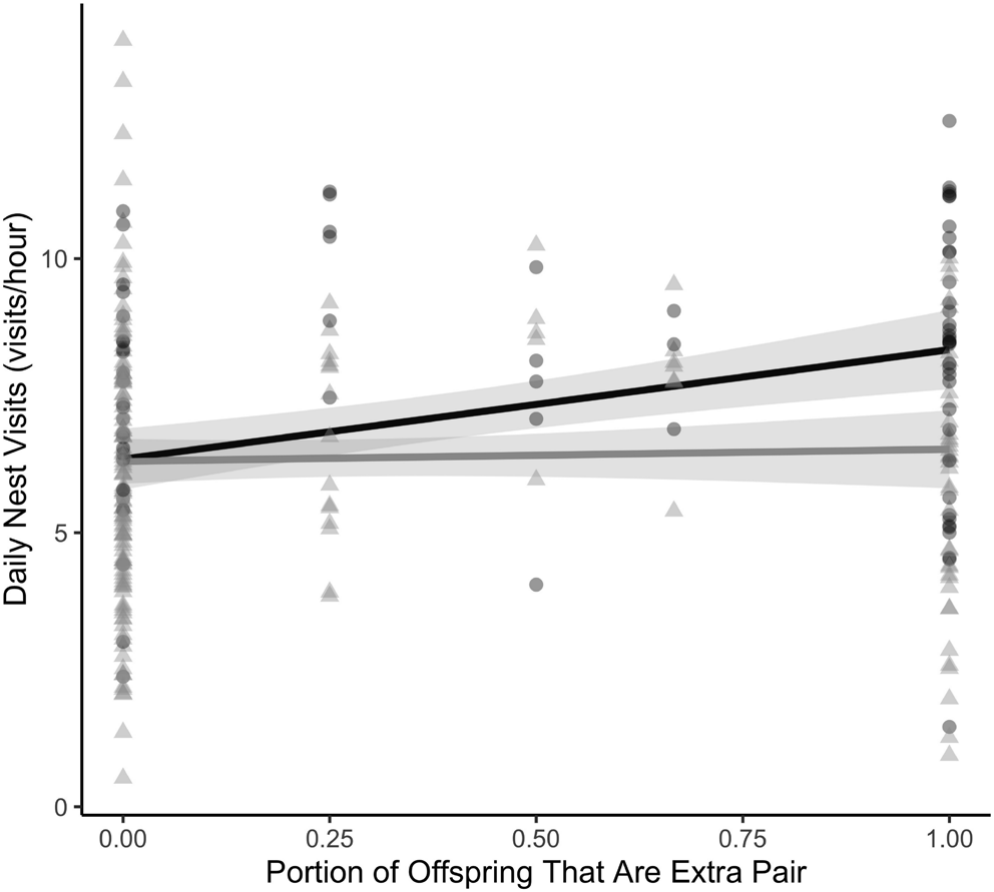
The relationship between maternal (black, circles) and paternal (gray, triangles) visitation rates and the proportion of extra‐pair young in the nest. Model predicted mean ± SE over raw data points, which each represents daily nest visitation rates for 12 females and 25 males.

We conducted statistical analyses using R (R Core Team 2023, v. 4.3.1). We determined how the presence or absence of EPY (0/1), the number of EPY (1–4), and the proportion of EPY (0–1) in the nest affected parental nest visitation rate using 3 separate linear mixed‐effects models (LMM). In each model, parental visitation rates (visit/h) for every day the nest was monitored was the response variable, and either presence (Model 1, Figure 1), number (Model 2, Figure 2), or portion (Model 3, Figure 3) of extra‐pair young, as well as parental sex were the predictor variables, with an interaction indicated, to explore if males and females were responding differently to EPY in the nest. Additionally, in all models, nestling age (0–10 days), Julian day, year, and the number of nestlings in the nest were included as fixed effect covariates. We then employed backward stepwise variable selection to only include variables with a *p*‐value of 0.2 or lower; we removed nestling age and year (Wang et al. 2007). Following this, all models included Julian Day and the number of nestlings in the nest as fixed effect covariates. The unique ID of each nest was included as a random effect to control for repeated measures of parental visitation across the nestling period and non‐independence of the behavior of social partners in the 19 nests for which we had measures of parental investment by both the male and female. Initally, nestling age was included to account for variation in monitoring duration (i.e., nest failure or difficulty capturing parents) and changes in parental care with nestling age. The day of the year was also included to account for sampling duration and seasonal changes in adult behavior. In all three models, we found significant interactions between our metric of extra‐pair paternity and paternal sex, which we explored further with a post hoc analysis. We fitted all LMMs using the package “lme4” (Bates et al. 2015) and checked the assumptions of LMMs using the “performance” package (Lüdecke et al. 2021), to which the models conformed. We tested the significance of fixed effects from the LMMs using the package lmerTest (Kuznetsova et al. 2017). Finally, we evaluated significant interactions between fixed effects with post hoc tests using the “emmeans” package (Lenth et al. 2019). Full model summaries for each model are included below (Tables 1, 2, 3).

**TABLE 1:** Results from Model 1.

Random effect	Variance	SD			
Nest ID	2.48	1.57			
Residual	3.17	1.78			

Fixed effects	Estimate	SE	Df	*t*	*p*
Cuckolded: Yes	2.27	0.83	41.86	2.74	0.009
Sex: Male	0.28	0.51	244.68	0.55	0.58
Day of Year	−0.03	0.01	47	−2.64	0.01
Brood Size	0.56	0.19	226.95	2.93	0.004
Cuckolded: Yes; Sex: Male	−1.93	0.63	244.44	−3.06	0.002

Contrast: Sex: Presence EPY	Estimate	SE	Df	*t* ratio	*p*
Female: No—Female: Yes	−2.27	0.83	46.8	−2.73	0.04
Female: No—Male: No	−0.28	0.51	244.7	−0.55	0.95
Female: No—Male: No	−0.62	0.82	44.1	−0.76	0.87
Female: Yes—Male: No	1.99	0.72	28.7	2.78	0.04
Female: Yes—Male: Yes	1.65	0.38	236.5	4.4	< 0.001
Male: No—Male: Yes	0.35	0.7	26.2	−0.49	0.96

*Note:* The results for the model exploring the correlation between parental visitation (Sex: Male/Female) and the presence/absence of EPY. The post hoc table shows the results from a pairwise comparison male and female behavioral responses to EPY young in the nest.

**TABLE 2:** Results from Model 2.

Random effect	Variance	SD			
Nest ID	2.46	1.57			
Residual	3.2	1.79			

Fixed Effects	Estimate	SE	Df	*t*	*p*
Number of EPY in the nest	0.86	0.37	41.23	2.33	0.02
Sex: Male	−0.04	0.44	231.26	−0.1	0.91
Day of Year	−0.03	0.01	49.03	−2.68	0.001
Brood Size	0.54	0.19	231.03	2.88	0.004
Number of EPY in the nest; Sex: Male	−1.05	0.36	170.2	−2.93	0.004

Contrast	Estimate	SE	Df	*t* ratio	*p*
Female—Male	1.05	0.37	176	2.88	0.005

*Note:* The results for the model exploring the correlation between parental visitation (Sex: Male/Female) and the number of EPY in the nest. The post hoc table shows the results from a pairwise comparison male and female behavioral responses to EPY young in the nest, and the predicted slopes.

**TABLE 3:** Results from Model 3.

Random effect	Variance	SD			
Nest ID	2.49	1.58			
Residual	3.2	1.79			

Fixed Effects	Estimate	SE	Df	*t*	*p*
Proportion of EPY in the nest	2	0.95	34.53	2.1	0.04
Sex: Male	−0.04	0.46	244.99	−0.09	0.93
Day of Year	−0.03	0.01	48.13	−2.67	0.01
Brood Size	0.57	0.19	225.38	2.99	0.003
Proportion of EPY in the nest; Sex: Male	−1.77	0.67	243.36	−2.66	0.008

Contrast	Estimate	SE	Df	*t* ratio	*p*
Female—Male	1.78	0.68	244	2.64	0.009

*Note:* The results for the model exploring the correlation between parental visitation (Sex: Male/Female) and the proportion of EPY in the nest. The post hoc table shows the results from a pairwise comparison male and female behavioral responses to EPY young in the nest, and the predicted slopes.

## 3 Results

We found that the nest visitation rate was influenced by the presence or absence of EPY in the nest and the sex of the social parent (*β*_EP:Male_ = 1.93 ± 0.63, *t*_243.44_ = −3.06, *p* = 0.002). Specifically, post hoc tests showed that males did not change their visitation rates based on the presence of EPY (*β*_Male_ = −0.34 ± 0.70, *t.ratio*_26.1_ = −0.48, *p* = 0.96), but females exhibited significantly higher visitation rates when EPY was present compared to when they were not (*β*_Female_ = 2.27 ± 0.83, *t.ratio*_46.80_ = 2.73, *p* = 0.04), independent of brood size and day of the year (Figure 1). Overall, this resulted in a significant effect of the presence (or absence) of EPY in the nest and nest visitation rates across the nestling period (*β*_EP:Yes_ = 2.27 ± 0.83, *t*_41.86_ = 2.74, *p* = 0.009): nests with EPY were visited more often than those without. Additionally, we found that nest visitation rates were positively associated within pairs (*β*_Male Visits_ = 0.50 ± 0.12, *t*_52.92_ = 4.22, *p* < 0.01) something previously observed in the focal population (Lane et al. 2023).

We found similar results when examining if and how the number and proportion of EPY in a nest were associated with the visitation rate. As above, we found that the sex of the parent mattered, whether examining the number of EPY (*β*_EP:Male_ = −1.05 ± 0.36, *t*_170.20_ = −2.93, *p* = 0.004; Figure 2) or the proportion of EPY (*β*_EP:Male_ = 1.77 ± 0.67, *t*_243.36_ = −2.66, *p* = 0.008; Figure 3) in a nest. Female nest visitation rates were positively associated with increasing number and proportion of EPY (slope = 0.86 ± 0.37; slope = 2.00 ± 0.95, respectively) whereas male nest visitations did not differ with the number or proportion of EPY in the nest (slope = −0.19 ± 0.37; slope = 0.22 ± 0.95, respectively). Overall, there was a significant, positive association between both the number (*β*_Number of EPY_ = 0.86 ± 0.37, *t*_41.23_ = 2.33, *p* = 0.02) and the proportion (*β*_Proportion of EPY_ = 2.00 ± 0.95, *t*_34.53_ = 2.74, *p* = 0.04) of EPY in the nest and nest visitation rate.

## 4 Discussion

The theoretical frameworks surrounding extra‐pair mating emphasize potential benefits to females (Westneat and Stewart 2003), yet comparatively few empirical studies have examined female behavioral responses to the presence of extra‐pair young. Rather, most studies examining the parental behavioral response to EP paternity in the nest have focused on males (Whittingham and Dunn 2001; Søraker et al. 2023). When females are studied, there can be an implicit assumption that their behavior is a compensatory response to male or helper behavior rather than independent modulation of their investment in a nest based on the potential benefits of EPY. In the current study, we examined how parental investment by both sexes varied with the presence of EPY by measuring nest visitation rates in a socially monogamous wild songbird. We found that female song sparrows with EPY in their nests visited significantly more often than did females without EPY (Figure 1), and that maternal visitation increased with the number (Figure 2) and proportion (Figure 3) of EPY in the nest. Conversely, we found no evidence that paternal visitation varied with any metric of EPY (Figures 1, 2, 3).

This study contributes to our understanding of the fitness costs and benefits of EP mating by examining the relationship between the presence of EPY and parental investment by both sexes. Our finding that female song sparrows increase visitation in response to the presence of EPY agrees with four previous studies that found increased maternal investment (egg volume, McFarlane et al. 2010; feeding rate, Chuang‐Dobbs et al. 2001; Suter et al. 2009; Sakamoto et al. 2023) in EP nests. Some previous studies (Chuang‐Dobbs et al. 2001; Suter et al. 2009) attributed variation in maternal care to female compensation for male reductions in care. However, in our population and this data set, male and female care within pairs were positively correlated (Lane et al. 2023) and males in this study did not reduce visitation, suggesting that the increase in maternal care with the presence of EPY is independent modulation of behavior, not compensation. In contrast to our findings, a study in an island population of song sparrows found that females did not change their feeding rate with the proportion of EPY (Gow et al. 2019). This contrast may be due to differences in the social mating system and environmental context of our song sparrow population. The island population had socially polygynous males that had higher proportions of EPY in their primary nest when compared to socially monogamous males (Gow et al. 2019), whereas we have not observed socially polygynous males in our mainland population. Further, two studies found reduced maternal investment (feeding rate, (Li and Brown 2002; Du et al. 2015)) with the presence of EPY in ground tits ( *Pseudopodoces humilis* ) and Mexican jays ( *Aphelocoma ultramarina* ) possibly due to increased feeding rates by the social or EP male at EP nests, respectively. Four other studies did not document an association between maternal feeding rate and the presence of EPY (Dixon et al. 1994; García‐Vigón et al. 2009; Maguire and Safran 2010; Li et al. 2021). Many others have examined male care in association with EPY, and though some have found that males reduce (Morton et al. 1990; Dixon et al. 1994; Chuang‐Dobbs et al. 2001; Suter et al. 2009; Gow et al. 2019) or increase (García‐Vigón et al. 2009; Du et al. 2015) care for nests with EPY, many align with our finding of no association between paternal investment and genetic paternity (Whittingham and Lifjeld 1995; Yezerinac et al. 1996; MacDougall‐Shackleton and Robertson 1998; Peterson et al. 2001; Li and Brown 2002; Dickinson 2003; Maguire and Safran 2010; Villavicencio et al. 2014; Barati et al. 2018; Cousseau et al. 2020; Gao et al. 2020; Li et al. 2021; Poblete et al. 2021). Our results and those of previous studies suggest that males of socially monogamous bird species may not be able to assess paternity and/or that the risks of harming their offspring by withholding care from nests with potential EPY are too high (Whittingham et al. 1992; Westneat and Sherman 1993; Whittingham and Dunn 2001). Meanwhile, although females are less frequently the focus of parental care and EPY studies, our results indicate that they are capable of modulating parental care in relation to EPY.

Although there are multiple hypotheses for why female songbirds might select particular extra‐pair partners, none of the explanations that have been tested have been supported in song sparrows. Extra‐pair male song sparrows did not have traits preferred by females (e.g., larger song repertoire sizes), nor did EPY have greater genetic diversity (Hill et al. 2011). Moreover, there was no evidence that EPY had higher lifetime reproductive success; they, in fact, had lower fitness than within‐pair young (Sardell, Arcese and Keller, et al. 2012). Yet, the offspring of male EPY were found to have higher recruitment than the offspring of within‐pair young (Sardell, Arcese and Reid, et al. 2012), indicating that there may be benefits of additive genetic variation (Sardell, Arcese and Keller, et al. 2012; Sardell, Arcese and Reid, et al. 2012; Reid and Wolak 2018). Nonetheless, our finding of increased maternal investment in nests with EPY suggests that extra‐pair mating has some benefit to females, or that females with the resources to engage in EPCs also had the capacity to invest more in maternal care. It is also possible that female song sparrows receive direct benefits from their EP copulations, such as infertility assurance or access to resources from the EP male (Hill et al. 2011). Additional access to resources would allow females to invest more in their current reproductive attempts. EP sires in this species are predominantly neighboring males (O'Connor et al. 2006; Sardell et al. 2010) with few EP copulations from floater males (Sardell et al. 2010), making female access to the EP male's resources a potentially sizeable contribution to her fitness. Since EP sires are predominantly neighboring males, females could potentially benefit from the cooperative behaviors of EP males, such as nest defense.

Overall, our results highlight the importance of examining female investment in response to EP paternity. The historic and continuing focus on male behavioral responses to nests with EPY leaves out an important consideration of EP mating: how and why does the female respond? Female reproductive success also has the potential to change with the presence of EPY in the nest yet has often been overlooked. Mixed results across studies suggest that context is important for female modification of investment, and more studies across a broader range of avian species and environments will help to tease apart factors influencing female response to EP paternity. Importantly, maternal behavior needs to be considered independently of potential compensation for male or helper behavior. Comparing female responses across systems with identified benefits from EP paternity could help describe contexts where females benefit from increased investment in EP nests.

## Author Contributions

**Valerie N. Brewer:** conceptualization (lead), data curation (supporting), formal analysis (supporting), investigation (lead), methodology (lead), project administration (lead), writing – original draft (lead), writing – review and editing (lead). **Samuel J. Lane:** conceptualization (equal), data curation (equal), formal analysis (lead), investigation (equal), methodology (equal), project administration (supporting), writing – original draft (supporting), writing – review and editing (equal). **Isaac J. VanDiest:** conceptualization (equal), investigation (equal), methodology (equal), writing – review and editing (equal). **Karen E. Mabry:** conceptualization (equal), funding acquisition (equal), methodology (equal), project administration (equal), resources (equal), writing – review and editing (equal). **Kendra B. Sewall:** conceptualization (equal), formal analysis (equal), funding acquisition (equal), project administration (equal), resources (equal), writing – original draft (equal), writing – review and editing (equal).

## Conflicts of Interest

The authors declare no conflicts of interest.

## Data Availability

All data and associated code are freely available on Dryad (DOI: 10.5061/dryad.gqnk98szq).
